# Maskless and low-destructive nanofabrication on quartz by friction-induced selective etching

**DOI:** 10.1186/1556-276X-8-140

**Published:** 2013-03-27

**Authors:** Chenfei Song, Xiaoying Li, Shuxun Cui, Hanshan Dong, Bingjun Yu, Linmao Qian

**Affiliations:** 1 Tribology Research Institute, National Traction Power Laboratory, Southwest Jiaotong University, Chengdu, Sichuan Province, 610031, People’s Republic of China; 2 School of Metallurgy and Materials, The University of Birmingham, Birmingham, B15 2TT, UK

**Keywords:** Maskless, Nanofabrication, Friction-induced selective etching, Quartz, 81.16.Rf, 68.37.Ps

## Abstract

A low-destructive friction-induced nanofabrication method is proposed to produce three-dimensional nanostructures on a quartz surface. Without any template, nanofabrication can be achieved by low-destructive scanning on a target area and post-etching in a KOH solution. Various nanostructures, such as slopes, hierarchical stages and chessboard-like patterns, can be fabricated on the quartz surface. Although the rise of etching temperature can improve fabrication efficiency, fabrication depth is dependent only upon contact pressure and scanning cycles. With the increase of contact pressure during scanning, selective etching thickness of the scanned area increases from 0 to 2.9 nm before the yield of the quartz surface and then tends to stabilise after the appearance of a wear. Refabrication on existing nanostructures can be realised to produce deeper structures on the quartz surface. Based on Arrhenius fitting of the etching rate and transmission electron microscopy characterization of the nanostructure, fabrication mechanism could be attributed to the selective etching of the friction-induced amorphous layer on the quartz surface. As a maskless and low-destructive technique, the proposed friction-induced method will open up new possibilities for further nanofabrication.

## Background

By virtue of its excellent chemical and physical properties, quartz has been widely used in micro/nanoelectromechanical systems (MEMS/NEMS), such as piezoelectric sensors [1], biochips [2], optical sensors [3], etc. Traditional lithographic fabrication on a quartz surface includes a complex process of mask deposition, exposure, etching and mask removal [4,5]. As device dimension has been down to nanoscale, traditional lithography hardly provides feasible nanofabrication on the quartz surface because of its involute process and limited resolution [6].

Although electron beam lithography has a better resolution in nanofabrication [7], high-energy beam can cause undesirable amorphization on quartz [8]. By virtue of their high precision, proximal probe methods based on scanning tunnel microscopy and atomic force microscopy (AFM) have been employed to fabricate nanostructures [9-13]. For example, AFM was used to fabricate a series of nanoscale grooves on polymers, metals and semiconductors by mechanical cutting [9]. However, it is inappropriate for quartz surface because of crack generation during the cutting process on such hard, brittle materials [10]. Although nanostructures can be fabricated on conductive surfaces by an anodic oxidation process at a given voltage, it is invalid for an insulator surface of quartz [11]. Recently, hillock-like nanostructures can be fabricated on silicon and quartz surfaces by sliding a diamond tip with repeated scratching cycles under suitable low loads [12,13]. However, since such hillocks are mainly generated from mechanical deformation of substrates, possible lattice damages may form on the surface of the hillocks and reduce their mechanical properties [14]. Because the lattice damages are detrimental to the applications of quartz devices [15,16], it is imperative to develop a straightforward and low-destructive nanofabrication method for the quartz surface.

In the present study, a novel nanofabrication method to produce three-dimensional nanostructures on the quartz surface has been developed by low-destructive scanning on a target area and post-etching in a KOH solution. The capability of this nanofabrication method was demonstrated by various nanostructures including slopes, hierarchical stages and chessboard-like patterns. The etching rate of the scanned area was tested at various temperatures. To produce deeper structures, refabrication was attempted on the existing nanostructures. The fabrication mechanism was discussed based on Arrhenius fitting of the etching rate and transmission electron microscopy (TEM) characterization of the nanostructure. In brief, the low-destructive friction-induced nanofabrication method may shed new light on nanotechnology.

## Methods

**Figure 1:**
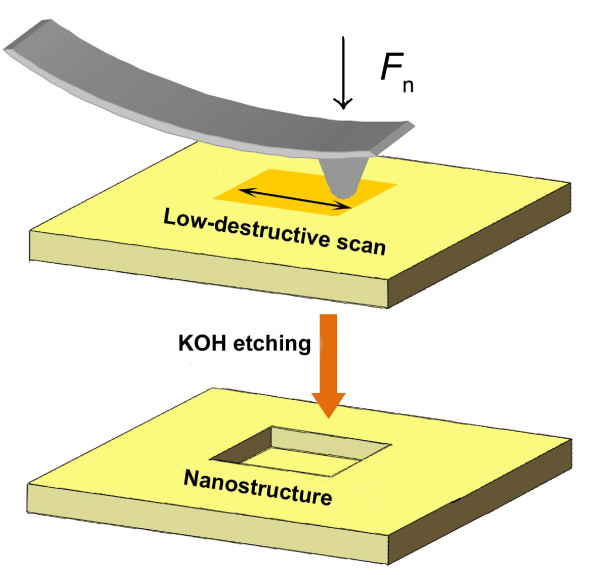
**Sketch map of the fabrication process.** Nanofabrication of quartz can be achieved by low-destructive scanning on a target area and post-etching in a KOH solution.

Monocrystalline quartz wafers (X-cut) with a thickness of 0.5 mm were purchased from Semiconductor Wafer, Inc. (Hsinchu, Taiwan). By AFM (SPI3800N, Seiko, Tokyo, Japan), the root-mean-square (RMS) roughness of the quartz wafers was measured as 0.15 nm over a 2 × 2 μm^2^ area. The whole fabrication process consists of two steps: low-destructive scanning on the target area and selective etching in the KOH solution, as shown in Figure 1. Low-destructive scanning was performed by AFM mounted with a diamond tip (Micro Star Technologies, Huntsville, TX, USA). The nominal curvature radius of the tip was 350 nm, and the spring constant of the cantilever was calibrated as 200 N/m [17]. To avoid the wear of surface, the applied normal load *F*_n_ was lower than 15 μN, under which the corresponding maximum Hertzian contact pressure was below 5.1 GPa [18,19]. After scanning, the demanded nanostructures were produced at the scanned area of the quartz surface by etching the samples in 20-wt.% KOH solution for appropriate periods at 293 K if not specially mentioned. A variety of nanostructures can be fabricated through the control of scanning load and tip traces. To improve the fabrication efficiency, quartz samples were etched at various temperatures ranging from 273 to 328 K after scanning under constant loads of 5, 8 and 12 μN. To get a deeper fabrication depth, refabrication was realised on an existing 5 × 5 μm^2^ area with a depth of 1.2 nm. All the AFM images of the nanostructures were scanned by Si_3_N_4_ tips with a nominal spring constant of 0.1 N/m (MLCT, Bruker Corp., Billerica, MA, USA).

To understand the mechanism of the low-destructive friction-induced nanofabrication, the microscopic structure of the scanned area of the quartz sample was detected by cross-sectional TEM (XTEM, JEOL JEM-2100 LaB6, JEOL Ltd., Tokyo, Japan) before and after KOH etching. The XTEM samples were prepared using a Quanta 3D FEG focused ion beam (FIB, FEI Company, Hillsboro, OR, USA) miller from the scanned area on quartz. In order to facilitate the FIB cutting across the scanned area, linescratch areas in the length of 200 μm were produced on the quartz samples by a nanoscratch tester (CSM Instruments, Peseux, Switzerland) with a spheral diamond tip having a radius of 20 μm. The low-destructive area was scratched under the normal load of 45 mN (the corresponding Hertzian contact pressure is 4.9 GPa), and the groove in a depth of 23 nm was scratched under the normal load of 95 mN.

## Results and discussion

### Results

#### Typical nanostructures produced on quartz by friction-induced selective etching

**Figure 2:**
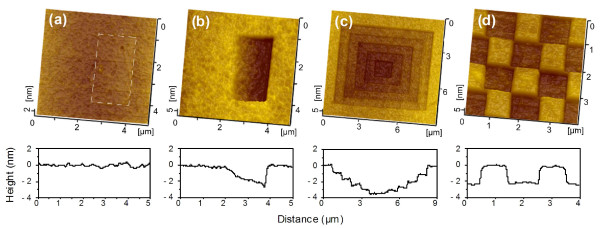
**Maskless and low-destructive nanofabrication on quartz. (a)** Low-destructive fabrication after scanning in 1.5× 3 μm^2^ area (line-marked) under progressive loads that ranged from 0 to 15 μN. **(b)** A slope produced by etching the area (a) in KOH solution for 3 h. **(c)** Five-step hierarchical structure produced by low-destructive scanning under *F*_n_ = 5 μN and etching for 3 h, where the scan size of each cycle was successively set as 7, 5.6, 4.2, 2.8 and 1.4 μm. **(d)** Chessboard-like patterns produced by low-destructive scanning on the selected area under *F*_n_ = 12 μN and etching for 3 h, where the scan size was 1 μm and the interval between adjoining scan centres was 2 μm.

Figure 2 shows the AFM images of a wearless surface and typical nanostructures produced by friction-induced selective etching. The 1.5 × 3 μm^2^ marked area in Figure 2a was scanned under progressive loads from 0 to 15 μN. Since no deformation or removal of material was observed on the scanned area, the scanning can be considered as a wearless process [20]. Unlike the mechanical cutting, this wearless scanning can avoid severe mechanical deformation or the generation of crack on the fabrication area. After etching for 3 h in KOH solution at 293 K, the material in the scanned area was selectively removed to form a slope, where the fabrication depth *D* increased from 0 to 2.9 nm with the increase of normal load *F*_n_ from 0 to 15 μN, as shown in Figure 2b. Since the superficial layer on the fabrication area was selectively etched, possible mechanical destruction on the fabrication area could be further reduced.

When the scan size was adjusted during repeated scanning, the hierarchical structure (as shown in Figure 2c) with five stages can be fabricated. Here, under a constant load of 5 μN and with a fixed centre point, the scanned area was successively set as 7 × 7, 5.6 × 5.6, 4.2 × 4.2, 2.8 × 2.8 and 1.4 × 1.4 μm^2^. Consequently, the overlapped areas were scratched by repeated scans. After selective etching for 3 h, the hierarchical structure was created, where the fabrication depth *D* of the stages increased from 1.2 to 3.5 nm with the increase of the number of repeated scanning cycles from 1 to 5. By programming the tip trace, the demanded patterns can also be produced at a target area. The chessboard-like patterns in Figure 2d were produced by *F*_n_ = 12 μN and *N* = 1. The scan size was 1 μm, and the interval between adjoining scan centres was 2 μm. After etching for 3 h, the separated convexity with 2.1 nm in height was formed.

#### Effect of etching temperature on fabrication efficiency

**Figure 3:**
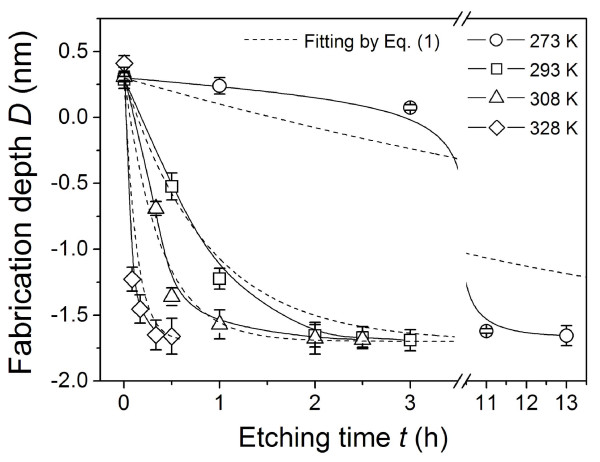
**Effect of etching temperature on fabrication efficiency.** The etching temperatures were set as 273, 293, 308 and 328 K. The quartz samples were scanned under the same normal load of 8 μN. Dashed lines were fitted by Equation 1.

Since all the mentioned fabrications were conducted at room temperature (293 K), it took about 3 h to finish selective etching of the scanned areas. To improve the efficiency of fabrication, selective etching of the scanned area was tested at various temperatures. As shown in Figure 3, the fabrication depth *D* versus etching time *t* curves showed a similar trend at different temperatures *T*, that is, keeping quasi-linear at the beginning and slowing down after a turning point. Such variation can be described by the following equation:

(1) $$D=1.9\times 8.83^{t}\exp\left[1.81t\times T/\left(2.87T-1000\right)\right]-1.6.$$

It was found that Equation 1 can fit *D* ~ *t* curves well when *T* is above 293 K. Even for *T* = 273 K, the fitting curve by Equation 1 shows a similar variation trend as the measured one.

The results in Figure 3 also suggested that different from the continuous etching in traditional lithography, the selective etching of the scanned area mainly occurred before the turning point. After the turning point in each curve, the etching rate was very low and the etching depth was quite close to the final fabrication depth. Therefore, a linear fit was used for estimating the average etching rate *η* before the turning point in each curve. Clearly, the rise of etching temperature can significantly improve the etching rate. With the increase of temperature from 273 to 328 K, the etching rate rose from 0.17 to 7.0 nm/h under the scan load of 8 μN. However, since etching pits appeared on the original surface at the etching temperature of 328 K in this experiment, the highest etching temperature for efficiency improvement of fabrication should be limited to 328 K. It was also noted that even though the etching rate *η* varied dramatically with temperature, the final fabrication depth *D*_*f*_ only relied on the scan load or the contact pressure. For instance, *D*_*f*_ was always about 1.6 nm for *F*_n_ = 8 μN regardless of the etching temperatures. The results have implied that the fabrication is indeed friction-controlled.

#### Refabrication on the existing nanostructures

**Figure 4:**
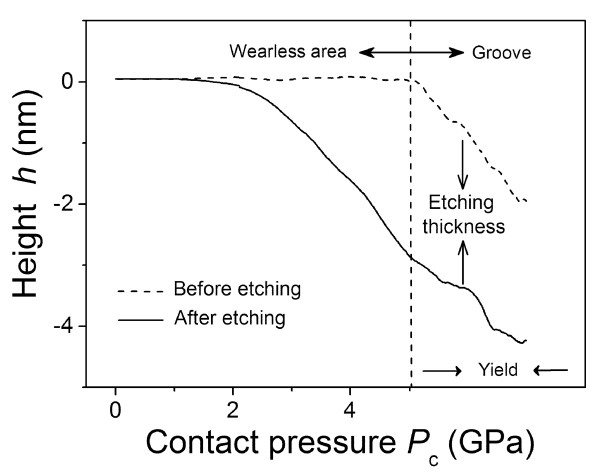
**Effect of contact pressure on the height of the fabrication area.** The height *h* of the fabrication area after scanning under progressive contact pressure *P*_c_. Dashed line, before KOH etching; solid line, after KOH etching.

**Figure 5:**
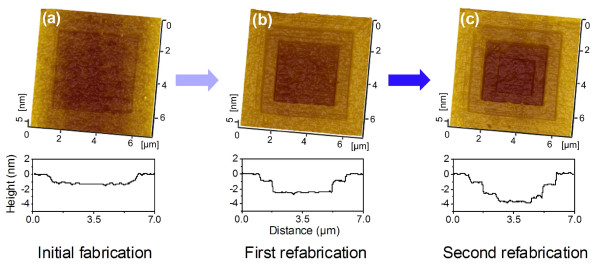
**Refabrication on an existing structure of quartz surface. (a)** Initial fabrication by low-destructive scanning under 5 μN on a 5 × 5 μm^2^ area and etching for 3 h at 293 K. **(b)** First refabrication by low-destructive scanning under 8 μN in the central 3.5 × 3.5 μm^2^ area and etching for 3 h at 293 K. **(c)** Second refabrication by low-destructive scanning under 5 μN in the central 2 × 2 μm^2^ area and etching for 3 h at 293 K.

Results showed that there was a critical fabrication depth after single low-destructive scanning and post-etching (Figure 4). To produce deeper structures in a low-destructive way, refabrication was attempted at target areas on existing nanostructures. Firstly, a 5 × 5 μm^2^ nanostructure with a depth of 1.2 nm was produced by wearless scanning under *F*_n_ = 5 μN and selective etching (Figure 5a). The first refabrication was carried out by wearless scanning under 8 μN in the central 3.5 × 3.5 μm^2^ area, and the additional fabrication depth was 1.6 nm after etching (Figure 5b). The second refabrication was finished by wearless scanning under 5 μN in the central 2 × 2 μm^2^ area, and the added depth was 1.1 nm (Figure 5c). Lastly, the final fabrication depth in the central 2 × 2 μm^2^ area was about 3.9 nm. Therefore, refabrication can provide a feasible way for producing a deeper structure on quartz surface in nanoscale.

### Discussion

#### Effect of contact pressure on low-destructive nanofabrication

From Figure 2b, it was noted that the fabrication depth increased with the increase of the scanning load or the contact pressure on the wearless scanned area. To analyse the effect of contact pressure on nanofabrication in a more detailed manner, a scratch consisting of a wearless area and groove was performed on the quartz surface under progressive loads from 0 to 40 μN by AFM. As shown in Figure 4, the cross-sectional profile of the scanned area (dashed line) revealed that the surface was wearless below *F*_n_ = 15 μN, where the corresponding contact pressure *P*_c_ was below 5.1 GPa. With the further increase of the normal load from 15 to 40 μN, the groove was formed on the surface and the wear depth increased from 0 to 1.7 nm (dashed line). After etching for 6 h at 293 K, the etched thickness increased from 0 to 2.9 nm on the wearless scanned area and then tended to stabilise on the groove. Therefore, the upper limit of the contact pressure for low-destructive scanning was 5.1 GPa, and the critical fabrication depth was 2.9 nm after single low-destructive scanning and post-etching. The fabrication of deeper nanostructure can be realised through repeated scanning or refabrication process (Figures 2c and 5).

From the results in Figure 3, it seemed that the eventual fabrication depth was only dependent on the contact pressure during the wearless scanning process, regardless of the etching temperature. To verify it, the fabrication depths under various contact pressures and etching temperatures were tested, and the results were summarised in Table 1. It was found that under the same contact pressure, the fabrication depths were almost the same for various etching temperatures. With the increase of normal load from 5 to 12 μN (the corresponding *P*_c_ from 3.5 to 4.7 GPa), the fabrication depth increased from 1.2 to 2.1 nm. Clearly, the contact pressure *P*_c_ was the decisive factor for the eventual fabrication depth at various etching temperatures.

**Table 1:** Fabrication depths under various scanning loads and etching temperatures (nm)

**Scanning load (μN)**	**273 K**	**293 K**	**308 K**	**318 K**	**328 K**
5	1.21	1.20	1.15	1.22	1.16
8	1.62	1.65	1.64	1.61	1.67
12	2.01	2.13	2.05	2.05	2.15

In brief, the contact pressure played a significant role in the low-destructive friction-induced nanofabrication. With the increase of contact pressure, the fabrication depth increased from 0 to 2.9 nm before the yield of quartz surface. Since the superficial layer of the scanned area can be etched selectively, it was reasonable to speculate that such layer may reveal a unique etching behaviour and microstructure. To understand the fabrication mechanism of the proposed method, it is essential to analyse the reaction kinetics of selective etching and detect the microstructures of the scanned area.

#### The mechanism of the low-destructive nanofabrication

**Figure 6:**
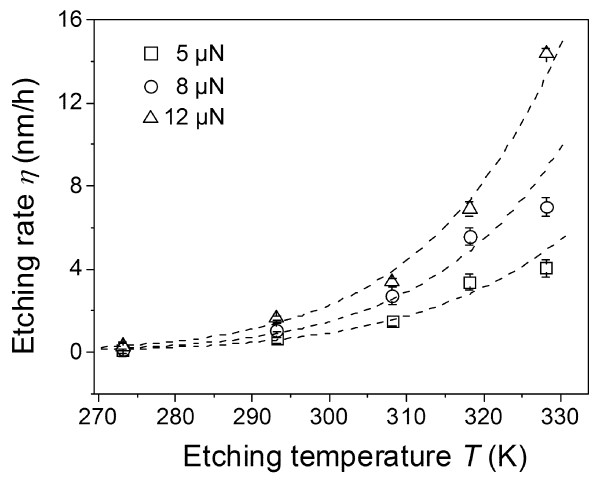
**Effect of normal loads on the etching rates at different etching temperatures.** Etching rates *η* of scanned area produced under various normal loads of 5, 8 and 12 μN at different etching temperatures. The corresponding contact pressure was 3.5, 4.4 and 4.7 GPa, respectively. Dashed line is Arrhenius fitting of the etching rates.

As shown in Figures 2 and 3, the proposed fabrication method was mainly based on the friction-induced selective etching on the scanned area. The etching reaction of quartz in KOH solution can be expressed as follows:

(2) $${\mathrm{SiO}}_{2}+2{\mathrm{KOH}=K}_{2}{\mathrm{SiO}}_{3}+H_{2}O,$$

which is a nucleophilic reaction. Based on chemical kinetics [21,22], the etching rate *η* can be described as follows:

(3) $$\eta =A{\left(c\right)}^{\alpha }\exp\left(-E_{a}/\mathrm{RT}\right),$$

where *A* is the frequency factor, *c* is the concentration of reactants, *α* is the reaction order, *R* is the gas constant, *T* is the etching temperature and *E*_a_ is the activation energy. Under a given etching temperature, the etching rate *η* was dependent only on *A*(*c*)^*α*^ and *E*_a_. To understand the role of contact pressure *P*_c_ in the friction-induced selective etching, the values of *E*_a_ and *A*(*c*)^*α*^ under various *P*_c_ were estimated through fitting the experimental *η ~ T* curves in Figure 6 by Equation 3 (dashed line).

As shown in Table 2, it was found that the contact pressure *P*_c_ had limited effect on the activation energy *E*_a_ but induced a large variation on the value of *A*(*c*)^*α*^. Since *α* is a constant in this erosion reaction, the dependence of the etching rate *η* on the contact pressure *P*_c_ should be mainly attributed to the variation of frequency factor *A* and the concentration of reactants *c*. As a stable oxide, there should be no further componential changes during the scanning process on the quartz surface. It was reported that the microscopic structure of solid had a significant effect on the chemical kinetics of fluid–solid reactions [23]. Therefore, it is logical to assume that the selective etching behaviours may be attributed to the structural changes of the scanned area on the quartz surface.

**Table 2:** **Fitting results in Figure**6

***F***_**n**_** (μN)**	***E***_**a**_** (kJ/mol)**	***A*(*c*)**^***α***^** (nm/h)**
5	48.3	2.3×10^8^
8	52.0	1.7×10^9^
12	51.3	2.1×10^9^

**Figure 7:**
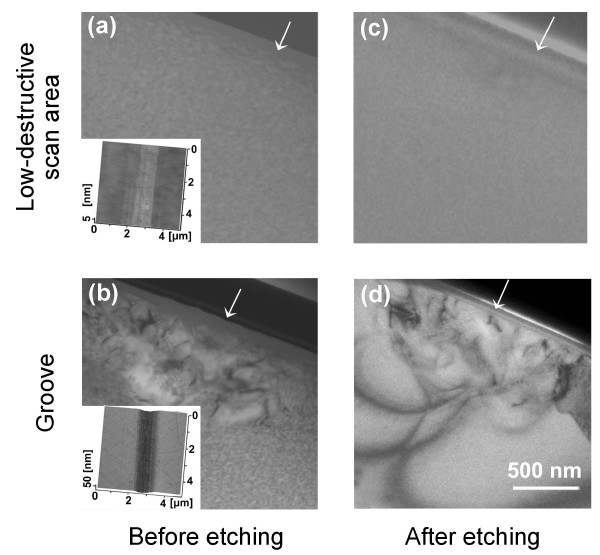
**XTEM observation of quartz samples produced by a nanoscratch tester.** TEM images of **(a)** low-destructive scanned area and **(b)** groove before KOH etching. TEM images of **(c)** low-destructive scanned area and **(d)** groove after KOH etching. The centre of each scanned area was marked with a white arrow. The inset pictures are the AFM images of the low-destructive scanned area and groove, respectively.

To further understand the fabrication mechanism of the proposed method, an XTEM observation was conducted on scratched quartz samples to detect the microscopic structures of the scanned area. Before KOH etching, the TEM observation revealed no visible deformation under the wearless scanned area (Figure 7a), while severe lattice distortion was observed under the groove (Figure 7b). After etching for 3 h at 293 K, there was still no visible damage on the wearless scanned area (Figure 7c), which further confirmed that the proposed method provided a low-destructive way for nanofabrication on quartz. At the same time, the distortion zone below the groove cannot be etched by KOH solution (Figure 7d). Combined with the results in Figure 4, because the formation of lattice distortion under groove did not help to increase the etching thickness, the selective etching of quartz might be related to other structural changes in the scanned area.

**Figure 8:**
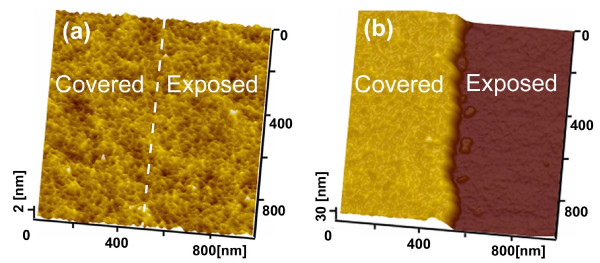
**Comparison of etching results on monocrystalline quartz and amorphous SiO**_**2**_**. (a)** No difference between covered surface and exposed surface on monocrystalline quartz after etching for 4 h at 293 K. **(b)** Exposed surface of amorphous SiO_2_ was etched evidently at 293 K.

Since amorphization usually occurs on the quartz surface during scratching and indentation [24,25], it is reasonable to speculate that amorphization during scanning/scratching at the superficial layer might have contributed to the selective etching of the quartz surface. To verify it, the etching rates of bulk amorphous SiO_2_ and crystal quartz were tested at 293 K. Before the tests, a gold film about 300 nm in thickness was deposited on half of each sample surface as a mask layer. After etching for 4 h in KOH solution and removal of the gold layers, there was no etching difference between the covered and exposed surfaces on the crystal quartz surface (Figure 8a). In contrast, a distinct step of 24 nm in height was formed on the amorphous SiO_2_ surface (Figure 8b). This has clearly demonstrated that amorphization can increase the etching rate of SiO_2_ in KOH. However, as the etching rate of the scanned area (about 1.65 nm/h at 4.7 GPa or 12 μN) was much lower than that of the bulk amorphous SiO_2_ (about 6 nm/h), it is most probably that the amorphization of the quartz surface after scanning under 4.7 GPa would be incomplete. Nevertheless, the friction-induced amorphization could be an acceptable explanation to the selective etching of the quartz surface although it is difficult, if not impossible, to clearly show the extremely thin (0 to approximately 3 nm), superficial and incompletely amorphized layer using the current XTEM technique and equipment.

Based on the mentioned discussions, the possible fabrication mechanism can be proposed as follows. In the KOH solution, the defective Si-O nets of friction-induced amorphous SiO_2_ can help the KOH solutes to preferentially diffuse into the scanned area and induce both higher concentrations of reactants *c* and a large number of colliding between reactants *A* in the scanned area [21,26]. According to the collide theory, the KOH etching of the scanned area depends on the total collision number between the KOH solutes and Si-O microscopic structure. Compared to the original surface, the scanned area has a faster etching rate and will be etched selectively. The etched depth may be decided by the thickness of the amorphous layer, and the etching rate is determined by the extent of amorphization. Higher contact pressure and repeated scanning can provide more gross energy *W* for the interaction between the tip and the quartz surface and consequently lead to faster etching rates and deeper structures. For example, under the conditions of *N* = 1 and *F*_n_ = 5 μN, the fabrication depth *D* was 1.2 nm and the etching rate *η* was 0.68 nm/h. The gross energy *W* dissipated during the scanning was calculated as 2.0 × 10^−9^ J [27]. To produce a deeper structure in depth of 1.6 nm, it needed 3.2 × 10^−9^ J by increasing *F*_n_ to 8 μN or 4.0 × 10^−9^ J by scanning one more cycle. At the same time, the etching rate *η* increased to 1.05 nm/h by increasing *F*_n_ to 8 μN, which meant that the increment of *η* was 0.31 nm/h per 10^−9^ J. As a comparison, the etching rate *η* increased to 1.21 nm/h by scanning one more cycle and the corresponding increment of *η* was 0.27 nm/h per 10^−9^ J. Therefore, compared to the number of scanning cycles, it seems that the scan load reveals a relatively stronger effect on the etching depth and etching rate. Nevertheless, the competitive relation between the load and scanning cycles should be investigated more thoroughly in the future.

In summary, a novel nanofabrication method based on friction-induced selective etching is proposed, by which three-dimensional nanostructures on demand can be created on a target quartz surface. This method enables nanofabrication more easily than photolithography with etching masks. This proximal probe technique makes it possible to fabricate at specified locations and to measure the dimensions of nanostructures with high precision. To overcome the critical etching thickness in a single fabrication flow, refabrication can be conducted on the existing structures. The friction-induced process under low contact pressure facilitates nanoscale material removal in a low-destructive way. Considering such advantages and potential applications, this method can open up new opportunities for future nanofabrication.

## Conclusions

In conclusion, we have presented a maskless and low-destructive method for nanofabrication on quartz based on AFM. Various nanostructures including slopes, hierarchical stages and chessboard-like patterns can be fabricated by changing the loading mode and programming the scan traces. Under the given experimental conditions, the surface of the scanned area is wearless below the critical contact pressure 5.1 GPa and the etched thickness of the scanned area goes up from 0 to 2.9 nm with the increase of contact pressure. Even though the rise of etching temperature can improve the efficiency of fabrication, the fabrication depth is controlled by the contact pressure and scanning cycles. Refabrication can be realised to overcome the critical etching thickness in a single fabrication flow. TEM observation shows that the formation of distortion does not contribute to the etching thickness. Analysis suggests that the fabrication mechanism could be attributed to the selective etching of friction-induced amorphous layer on the quartz surface. The proposed method based on friction-induced selective etching will provide new opportunities for nanofabrication on quartz surface.

## Abbreviations

AFM: Atomic force microscopy; FIB: Focused ion beam; TEM: Transmission electron microscopy; XTEM: Cross-sectional TEM.

## Competing interests

The authors declare that they have no competing interests.

## Authors’ contributions

CS and BY realised the fabrication experiments and acquired the original data in this paper. XL and HD did the TEM observation and analysis. LQ and SC have made substantial contributions to the concept and design of this paper. All authors read and approved the manuscript.
